# Modification of the nanostructure of lignocellulose cell walls via a non-enzymatic lignocellulose deconstruction system in brown rot wood-decay fungi

**DOI:** 10.1186/s13068-017-0865-2

**Published:** 2017-07-11

**Authors:** Barry Goodell, Yuan Zhu, Seong Kim, Kabindra Kafle, Daniel Eastwood, Geoffrey Daniel, Jody Jellison, Makoto Yoshida, Leslie Groom, Sai Venkatesh Pingali, Hugh O’Neill

**Affiliations:** 1 Department of Microbiology, Morrill Science Center IV, University of Massachusetts, Amherst, MA 01003-9298 USA; 2 State Key Laboratory of Microbial Resources, Institute of Microbiology, Chinese Academy of Sciences, Beijing, 100101 China; 3 Department of Chemical Engineering and Materials Research Institute, Pennsylvania State University, University Park, PA USA; 4 Department of Biosciences, Swansea University, Singleton Park Campus, Swansea, UK; 5 Department of Forest Products/Wood Science, Swedish University of Agricultural Science, Uppsala, Sweden; 6 Center for Agriculture, Food and the Environment, University of Massachusetts, 316 Stockbridge Hall, Amherst, USA; 7 Department of Environmental and Natural Resource Science, Tokyo University of Agriculture and Technology, Fuchu, Tokyo, Japan; 8 USDA Forest Service, Southern Research Station, Pineville, Louisiana 71360 USA; 9 Biology and Soft Matter Division, Oak Ridge National Laboratory, Oak Ridge, TN 37831 USA

**Keywords:** Chelator-mediated Fenton, Brown rot fungi, Small angle neutron scattering, Cellulose crystallinity, Lignin depolymerization, Biomass biorefinery

## Abstract

Wood decayed by brown rot fungi and wood treated with the chelator-mediated Fenton (CMF) reaction, either alone or together with a cellulose enzyme cocktail, was analyzed by small angle neutron scattering (SANS), sum frequency generation (SFG) spectroscopy, Fourier transform infrared (FTIR) analysis, X-ray diffraction (XRD), atomic force microscopy (AFM), and transmission electron microscopy (TEM). Results showed that the CMF mechanism mimicked brown rot fungal attack for both holocellulose and lignin components of the wood. Crystalline cellulose and lignin were both depolymerized by the CMF reaction. Porosity of the softwood cell wall did not increase during CMF treatment, enzymes secreted by the fungi did not penetrate the decayed wood. The enzymes in the cellulose cocktail also did not appear to alter the effects of the CMF-treated wood relative to enhancing cell wall deconstruction. This suggests a rethinking of current brown rot decay models and supports a model where monomeric sugars and oligosaccharides diffuse from the softwood cell walls during non-enzymatic action. In this regard, the CMF mechanism should not be thought of as a “pretreatment” used to permit enzymatic penetration into softwood cell walls, but instead it enhances polysaccharide components diffusing to fungal enzymes located in wood cell lumen environments during decay. SANS and other data are consistent with a model for repolymerization and aggregation of at least some portion of the lignin within the cell wall, and this is supported by AFM and TEM data. The data suggest that new approaches for conversion of wood substrates to platform chemicals in biorefineries could be achieved using the CMF mechanism with >75% solubilization of lignocellulose, but that a more selective suite of enzymes and other downstream treatments may be required to work when using CMF deconstruction technology. Strategies to enhance polysaccharide release from lignocellulose substrates for enhanced enzymatic action and fermentation of the released fraction would also aid in the efficient recovery of the more uniform modified lignin fraction that the CMF reaction generates to enhance biorefinery profitability.

## Background

Brown rot fungi in the three orders of Boletales, Gloeophyllales, and Polyporales are known to deconstruct wood in a highly efficient manner [1] using a non-enzymatic mechanism known as the chelator-mediated Fenton (CMF) system [2–5]. Woody plant biomass production and subsequent decomposition underpin the cycling and sequestration of carbon in forests systems and directly impact soil condition [6]. Wood is systematically depolymerized by microbial community succession that is dominated by brown rot species in conifer-rich forests [7]. Increasingly, research has focused on the interplay between substrate depolymerization and decay community structure and diversity, including priority effects and the evolution of decay modes [8, 9]. However, the role of non-enzymatic decomposition reactions in polymer-rich woody substrates by microbes is not widely reported compared to well defined enzyme-based decay mechanisms.

Prior research has demonstrated that while the polysaccharide components are initially depolymerized during brown rot primarily by the action of hydroxyl radicals generated via the CMF reaction [10–12], lignin is also depolymerized and then rapidly repolymerized by these radicals [13–16]. The in vitro action of hydroxyl radical treatment mimics the action of brown rot in early stages of wood degradation [12, 13]. Concurrent with the development of the CMF system, as brown rot fungi evolved independently multiple times from progenitor white rot fungi, they lost genes encoding lignin-degrading peroxidases and many of the genes associated with white rot holocellulose deconstruction. In most species, lignin-degrading phenoloxidases were also lost, but the GH5 cellulases and GH28 pectinases are increased. Concurrent with the loss of these enzymes, the brown rot fungi have developed a non-enzymatic mechanism to deconstruct the lignocellulose framework by catalytically modifying lignin and depolymerizing cellulose. Because of the complex nature of the lignin molecule and how it encrusts and intersperses with holocellulosic components, the lignin must be depolymerized and modified, and much of the depolymerized fraction then repolymerizes [13]. This process has been assumed to open the wood cell structure to allow greater access to enzymes during the later stages of decay; however, this aspect of the mechanism has not previously been demonstrated. The specific iron-reducing chelators involved in CMF chemistry appear to vary among fungal species, although a dominant hydroquinone, 2,5-dimethoxyhydroquinone (2,5-DMHQ) has been isolated from several species and shown to be capable of functioning as a redox cycling chelator [17, 18]. The production of a non-reducing chelator, oxalate, by the fungus, has also been demonstrated to be required for solubilization and sequestration of iron prior to reduction by the redox cycling chelator [4, 16, 17, 19].

The biochemistry of *Gloeophyllum trabeum*, an economically important brown rot fungus, has perhaps been studied more than any other brown rot fungus because of the ubiquitous decay that it causes, particularly in softwood species. In the current work, we examine the effect of non-enzymatic chemical decay reactions on wood, a complex polymer substrate, by comparing the action of *G. trabeum* to that of CMF treatment, using catalytic chemistries in the absence of the fungus. Another brown rot, *Rhodonia placenta*, is used in some assays for comparative purposes. In addition to conventional chemical analysis, we explore the use of small angle neutron scattering (SANS) and sum frequency generation (SFG) analyses, to better assess both how the nanoscale structure within wood changes and also how holocellulose crystallinity changes. For both SANS and SFG, this is the first time that either technique has been used to analyze decayed wood. Defining how the microbe-mediated chemical environment alters the structure of wood will provide insights into the process of decomposition, critical to understanding the interplay between enzymatic and non-enzymatic mechanisms and saprotrophic microbial community functioning in forests, but also providing insight into the application of these microbial chemistries for bioprocessing.

## Methods

### Wood shavings and chemicals

Wood shavings were machined from the sapwood of southern yellow pine (*Pinus* spp.) to a thickness range of 110–160 µm. An enzymatic cocktail Cellic CTec2 (a proprietary mixture of cellulase enzymes) was provided as a gift from Novozyme, and was used in this work where specified. The activity of the received Cellic CTec2 was 50–60 FPU/mL [20]. All other chemical reagents, including iron(III) chloride hexahydrate (FeCl_3_·6H_2_O), 2,3-dihydroxybenzoic acid (2,3-DHBA), 30 wt% hydrogen peroxide (H_2_O_2_), manganese chloride tetrahydrate (MnCl_2_·4H_2_O), and hydrogen chloride (HCl), sodium hydroxide (NaOH) were used as purchased from Thermo Fisher Scientific Company with no further purification.

### Small angle neutron scattering (SANS) analysis

#### Preparation of specimens for SANS analysis

##### *Gloephyllum trabeum* exposure of wood shavings

Wood shavings were submerged in distilled water for 2 h, drained, and then autoclaved in 500 mL acid-washed flasks. The flasks containing wood shavings were subsequently inoculated with *G. trabeum* mycelium from agar plates taking care to transfer minimal amounts of the agar, and then shaken to distribute the mycelium before incubating for either 18 or 42 days (2 replicates each), designated as 18dGt and 42dGt, respectively. Preparation of mycelial samples for use in generating SANS scattering data was done to provide bulk nano-to-mesoscale structural information on the fungal biomass apart from the wood material. This was achieved by growing *G. trabeum* mycelium for 20 days in Highley’s liquid medium [21] modified by adding 1 mM FeCl_3_·6H_2_O, and also by adjusting the pH to either 4 or 6.5 with HCl or NaOH, respectively. Mycelia were harvested by removal of the liquid media by filtration to maintain the extracellular matrix (ECM) glycoprotein sheath with the hyphae, and frozen until analysis.

##### Chelator-mediated Fenton (CMF) treatments

Iron, manganese and 2, 3-DHBA solutions (concentrations as described in Table 1) were prepared in acetate buffer (pH 4, 1.1 M). Wood shavings dried at 60 °C (0.75 g) were submerged in 25 mL of either one or both metal solutions, and then shaken for 10 min before drying at 120 °C for 2 h to remove free moisture. 25 mL 2,3-DHBA solution was then added, and the mixture was shaken for 30 min at room temperature. 25 mL of 0.5 M H_2_O_2_ was subsequently added and shaken at 40 °C and 125 rpm overnight (about 12 h) before decanting off the solution phase. The H_2_O_2_ addition was then repeated. After 32 h, the H_2_O_2_ was drained from the samples which were then frozen for later analysis (Table 1).

**Table 1:** CMF and cellulose enzyme treatments of wood samples used in SANS analysis

	Metal (50 mM)		2,3-DHBA (50 mM)	H_2_O_2_ (0.5 M)	Enzyme cellic CTec2
	Fe	Mn			
CMF(Fe)	+	−	+	+	−
CMF(Fe + Mn)	+	+	+	+	−
CMF(Mn)	−	+	+	+	−
CMF(Fe) + Enz	+	−	+	+	+
CMF(Mn) + Enz	−	+	+	+	+
Enz	−	−	−	−	+

##### Cellulase treatment

Cellic CTec2 (equivalent to 10–12 FPU/g wood in 50 mM acetate buffer, pH 5) was used to treat a portion of the wood shavings with CMF pretreatments, and these samples were then further shake-incubated for 12 h at 40 °C before oven-drying (105 °C) (Table 1).

All samples were saturated (4×) with 100% D_2_O to maximize D/H exchange and enhance scattering contrast before SANS experiments.

#### Instrument configuration and background on the use of Bio-SANS instrumentation

SANS is a technique that provides information in materials at length scales from 1 to 1000 nm [22]. The non-destructive and penetrating nature of neutrons enables studies of a wide range of materials ranging from macromolecules, polymers, colloids, porous systems, biological membranes, proteins, and other molecular assemblages. Diverse mesoscale structural information can be obtained including internal structure, particle concentration, and correlation between particles. SANS has previously been used to investigate the morphological changes in lignocellulose during chemical pretreatment and enzymatic digestion [23, 24], the structure of lignins in aqueous solution [25], and pH-dependent conformational changes in cellulases [26].

SANS data [27] were obtained on the Bio-SANS instrument at the High Flux Isotope Reactor (HFIR), Oak Ridge National Laboratory, USA. D_2_O-exchanged samples were densely loaded into titanium cells, which consisted of two quartz windows sandwiching a 0.5 mm thick aluminum spacer for sample loading. The cell windows were flanged with Viton o-rings and placed within a titanium holder that was screw-sealed. The cell was then filled with D_2_O via a cell port and all air bubbles removed. Scattering data were collected at sample-to-detector distances of 2.529 and 15.329 m to obtain data over a scattering vector range of 0.003 to 0.4 Å^−1^ using 6 Å neutrons. The scattering vector *Q*, (*Q* = 4(π)sin(*θ*)/*λ*) describes the relation of *Q* to lambda (neutron wavelength), and 2(*θ*), the scattering angle. Bio-SANS data reduction software implemented in an Igor Pro package (Wavemetrics) was used to generate 1 day scattering curves with corrections made for detector dark current (electronic noise), pixel sensitivity, and solvent-scattering backgrounds from D_2_O and the empty quartz cell [28]. SANS data were analyzed using the Unified Fit implementation of IRENA software in Igor Pro [29] at different stages of decay, as conducted previously for analysis of plant biomass SANS data [28]. Based on the structural features observed in the SANS profiles in our study, 3-levels were employed to extract structural organization in the low-*Q* (0.003 –0.006 Å^−1^), mid-*Q* (0.006–0.06 Å^−1^), and high-*Q* (0.06–0.3 Å^−1^) regions. For each of these three levels, a power-law exponent, *P* and/or a characteristic dimension *R*_g_, were extracted [30, 31] including their confidence range.

### SFG, XRD, and FTIR analysis

For exposure to the brown rot fungi *G. trabeum or R. placenta*, southern pine wood (*Pinus* spp.) was cut radially (quarter sawn) into thin wafers (10 mm square and 1 mm thick). The thin wafers were used rather than shavings because shavings, particularly when decayed, could not be easily place in sample holders for these analyses. Samples were saturated with distilled water and autoclaved prior to placement in soil-block chambers [32] and incubated with either fungus for 0, 10, 20, or 50 days. For CMF treatment, the wafers or southern pine wood shavings (0.5 mm thick) were treated with 1 mM FeCl_3_·6H_2_O, 1 mM DHBA, and 40 mM H_2_O_2_, with and without 1 mM oxalate and compared to samples treated with the same reagents in the absence of iron. A single pulse treatment was used to limit the degradation that occurred. Samples were incubated for 24 h at 40 °C before analysis. Oxalate was used in this work in part because it is known to be secreted by decay fungi as the attack of lignocellulose materials initiates, and brown rot decay fungi have been shown to solubilize and then recrystallize oxalate which has been suggested as a means to maintain pH control [33–35].

Vibrational sum frequency generation (SFG) spectroscopy analyses of fungal degraded wood wafers were conducted using a broadband SFG system that permitted femto-second broadband IR pulses and pico-second narrowband 800 nm pulses. Details of this SFG system have been described previously [36]. The probe volume of our SFG system was estimated to be about 120 × 80 μm wide over the sample and approximately 10–20 μm deep from the external surface of the sample [36].

XRD experiments were performed using a PANalytical Empyrean diffractometer (PANalytical, Netherlands) equipped with a Cu X-ray source (*λ* = 1.5404 Å) operated at 45 kV and 40 mA. Fungal degraded ground wood from the wafers was placed on a quartz zero-background holder before analysis. Scans were measured at 2*θ* in the range of 8–45° using a 0.05 step. The diffractograms were plotted with a constant y-offset for representative purposes.

Attenuated total reflectance Fourier transform infrared (ATR-FTIR) spectra of the same samples were collected using a Nicolet 8700 FTIR Spectrometer (Thermo Scientific) equipped with a smart iTR diamond ATR unit, a KBr beam splitter, and a deuterated triglycine sulfate (DTGS) detector. All spectra were collected from the wood wafer and ground wood samples in the region of 650–4000 cm^−1^ with a 4 cm^−1^ resolution and averaged over 100 scans. All spectra were normalized over 2930 cm^−1^ for presentation purposes.

### Atomic force microscopy (AFM) of brown-rotted wood surfaces

For the AFM work, *R. placenta* was used to decay southern pine blocks. Soil-block chambers as described in “Small angle neutron scattering (SANS) analysis” were used to decay pine blocks for approximately 8 weeks. The wood samples were then split along the radial surface, and the exposed S2 region of the wood cell walls was observed by AFM (Nanoscope IIIa AFM-Digital Instruments, Santa Barbara, California USA) with three 5 µm scans collected at each surface for comparative purposes. Control pine samples were imaged similarly. Images were obtained in intermittent-contact mode (tapping mode, TM) at a scan rate of 1 Hz. Three data channels—height, amplitude, and phase shift—were monitored during the image acquisition.

### Transmission electron microscopy (TEM) analysis

Samples of pine (*Pinus sylvestris* L.) and birch (*Betula verrucosa* Ehr.) wood (5 × 15 × 40 mm; along the grain) were incubated in agar plate cultures with *G. trabeum* for 8 and 10 weeks respectively. Birch wood was used as a reference/comparison to the pine in this work. Samples were removed from culture and sectioned with a razor to produce small 1 × 1 mm samples with an exposed cross-sectional face. These smaller samples were fixed in 3% v/v glutaradehyde containing 2% paraformaldehyde in 0.1 M sodium cacodylate buffer (pH 7.2) for 3 h. Following fixation, samples were washed in buffer (3 × 30 min), dehydrated in ethanol (20–100%; 20% step 20 min) and embedded in London white resin (London Resin Co, Basingstoke). Sections (90 nm) were cut using a Reichert ultramicrotome and stained with 2% w/v uranyl acetate. Sections were examined using a Philips CM12 TEM instrument (Philips, Eindhoven, The Netherlands) at 60 and 80 keV and images recorded on Kodak 4489 negative film and the films subsequently scanned using an Epson Perfection Pro 750 film scanner.

### Flow chart

**Fig. 1:**
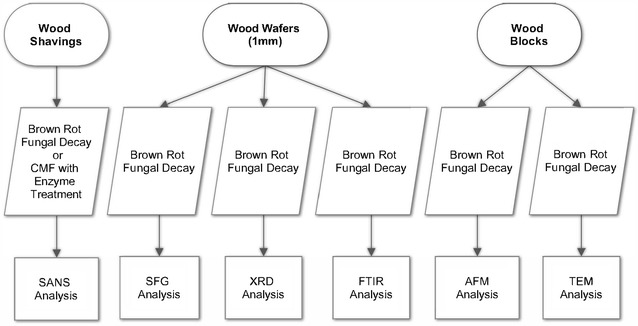
Flow chart detailing the materials, treatments and analyses conducted on samples for this research

Figure 1 is a schematic representation which summarizes the materials, methods, and analyses conducted as part of this research.

## Results

### Analysis of decayed samples

#### SANS analysis of fungal hyphae and decayed lignocellulose

The SANS scattering curves from the fungal hyphae and ECM grown in the absence of wood were featureless across low, medium, and high *Q* regimes, and for both pH conditions assayed (pH 4 and 6.5) little scattering interference was observed. The two pH values selected bracket the range that would be expected to occur within the decayed cell wall; however, the initial pH of wood can be driven down to as low as pH 2.0 by oxalate and other organic acids generated by the brown rot fungi in wood cell lumens. Since no significant SANS features were observed across the broad structural *Q* regime measured for the fungal biomass samples, this allowed subsequent SANS analysis of decayed wood to occur with minimal scattering contribution from fungal biomass.

**Fig. 2:**
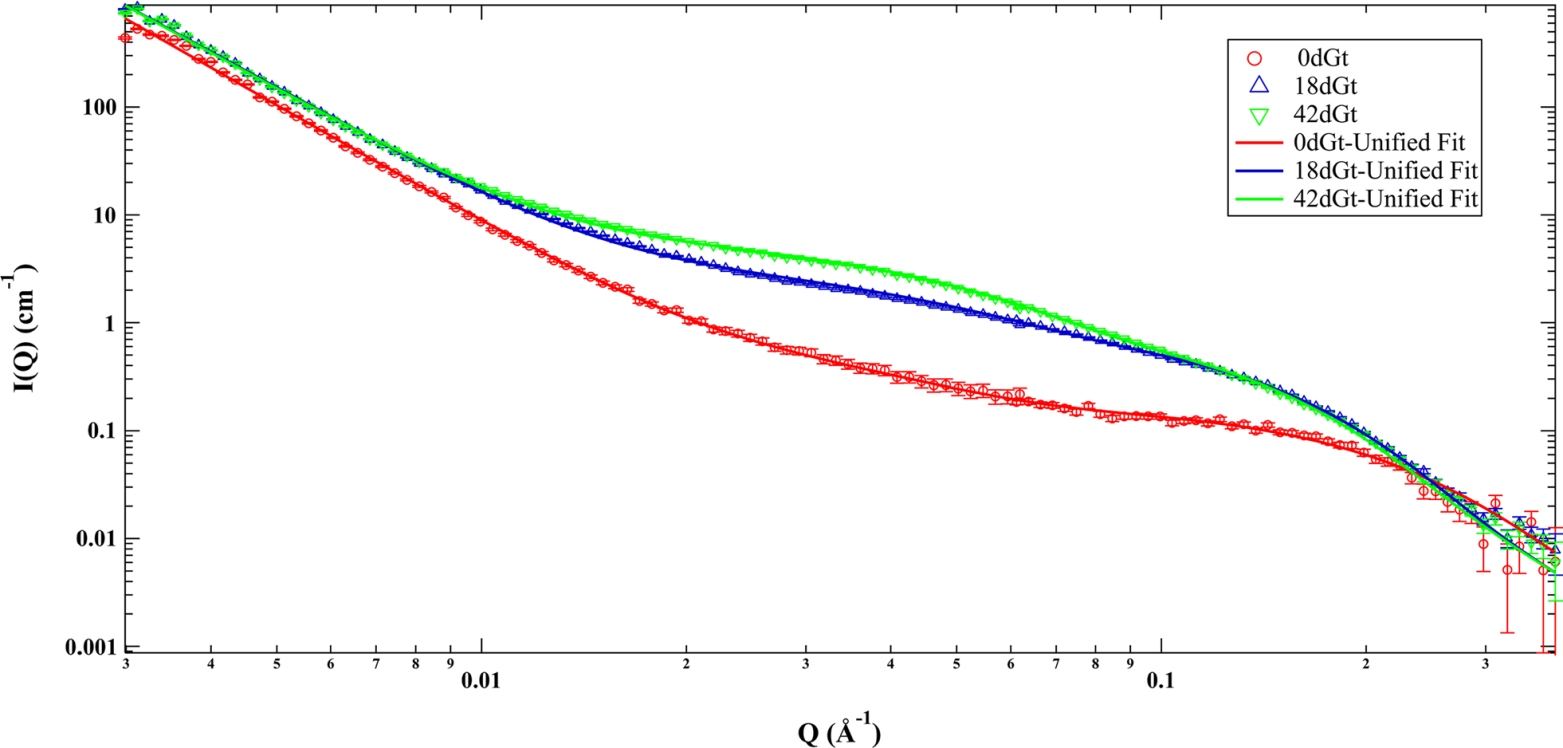
SANS profiles of thin wood shavings infected by *G. trabeum* for 0 (0dGt—control), 18 (18dGt), and 42 (42dGt) days. *I*(*Q*) is the measured scattered intensity and *Q* is the scattering wave vector

Comparison of SANS data from the decayed and control wood samples demonstrates that as decay by *G. trabeum* progressed over time significant structural changes occurred between 0.02 and 0.1 *Q* (Å^−1^) (Fig. 2) indicating that the most pronounced change in response to decay treatment occurred in the high- and mid-*Q* regimes. In the high-*Q* regime, a characteristic size parameterized as the radius of gyration, *R*_g_, increased from 9.10 for undecayed material to 13.50, and 14.40 Å for sample material exposed to *G. trabeum* for 18 and 42 days, respectively (Table 2).

**Table 2:** SANS structural parameters obtained from the unified fit analysis of samples at 0, 18, or 42 days decay treatment

Samples	High-*Q* (0.06–0.3 Å^−1^)		Mid-*Q* (0.006–0.06 Å^−1^)		Low-*Q* (0.003 –0.006 Å^−1^)
	*R*_*g*_ (Å)	*D* (cylindrical cross-section diameter Å)	*R*_*g*_ (Å)	*D* (Å) (spherical diameter)	*P*
0dGt	9.1 ± 1.3	21 ± 3	–	–	3.63 ± 0.1
18dGt	13.5 ± 0.1	31.2 ± 0.1	39 ± 3	100 ± 8	3.5 ± 0.1
42dGt	14.4 ± 0.6	33 ± 1	38.3 ± 1.50	99 ± 4	3.5 ± 0.1

*R*_g_ radius of gyration, *D* diameter, *P* power-law exponent

In previous studies, the *R*_g_ in the high-*Q* regime has been associated with the cross-sectional dimension of the cellulose elementary fibril [28, 37]. Our current data suggest that attack of the cellulose elementary fibril surfaces occurs initially in the decay process with removal of the more amorphous cellulose at the surface. As this depolymerization and solubilization progresses, it is then followed by a coalescence of adjacent microfibrils. In the initial stages of decay, the crystalline core of the elementary fibrils would remain intact, but as decay progressed through to the 42-day period, the core of the crystallites would also ultimately be depolymerized. This process would be facilitated by solubilization and removal (diffusion) of hemicellulose and the disruption and subsequent repolymerization of lignin. SANS data indicate that significant structural changes occurred over time in the samples in the first 18 days and after this time point, the SANS curves are similar over the measured *Q*-range except for a small increase in intensity in the mid-*Q* region. This indicates that most of the degradation occurred (or the degradation process is largely complete), under the experimental conditions used, within the first 18 days and then smaller incremental changes in the cellulose structure occur after this time, up to 42 days. This supports previous work showing that brown rot organisms attack wood by rapidly depolymerizing lignin and holocellulose with subsequent metabolism of the sugar and oligosaccharide residues [3, 12–14, 38, 39]. Based on knowledge of the compositional data combined with the SANS data presented here, we postulate that the high-*Q R*_g_ increase observed was related to the development of partially degraded cellulose microfibrils, which then coalesced. This would also be consistent with a moderate loss in glucose and concomitant partial loss of cellulose crystallinity (“SFG, XRD and FTIR analysis of fungal decayed lignocellulose”), together implying that the elementary fibril structure erodes as decay progresses.

As decay by *G. trabeum* progressed, an effective radius of gyration, *R*_g_, in the mid-*Q* region was observed after 18 days, and this does not change as decay progressed to the 42 day time point. Changes in the 0.015–0.08 Å^−1^ region correspond to features of 8–40 nm in the sample, and we relate this to redistribution of lignin into globular deposits within the wood cell wall. As reviewed in the Introduction, depolymerization and rapid repolymerization of lignin is known to occur as decay progresses, and under the decay conditions used in our research, the SANS data suggest that the majority of this lignin modification and aggregation occurred within the first 18 days of decay by *G. trabeum*. We posit that this reflects how lignin aggregates grow in size as decay by brown rot progresses to form these repolymerized and redistributed lignin deposits (see also “Atomic force microscopy (AFM) of brown rotted wood surfaces” on AFM observations). Scattering features associated with changes in lignin in the mid-*Q* region (*R*_g_ = 50–100 Å) have been previously reported in lignified wood samples, and changes similar to those observed in our work in the mid-*Q* region have been associated with increasing lignin aggregation [40]. The low-*Q* region is similar in all scattering curves as indicated by the similarity in the exponent of the power-law (see Table 1), indicating that the surface morphology of the cell walls at the angstrom level is largely unchanged during degradation.

#### SFG, XRD, and FTIR analysis of fungal decayed lignocellulose

**Fig. 3:**
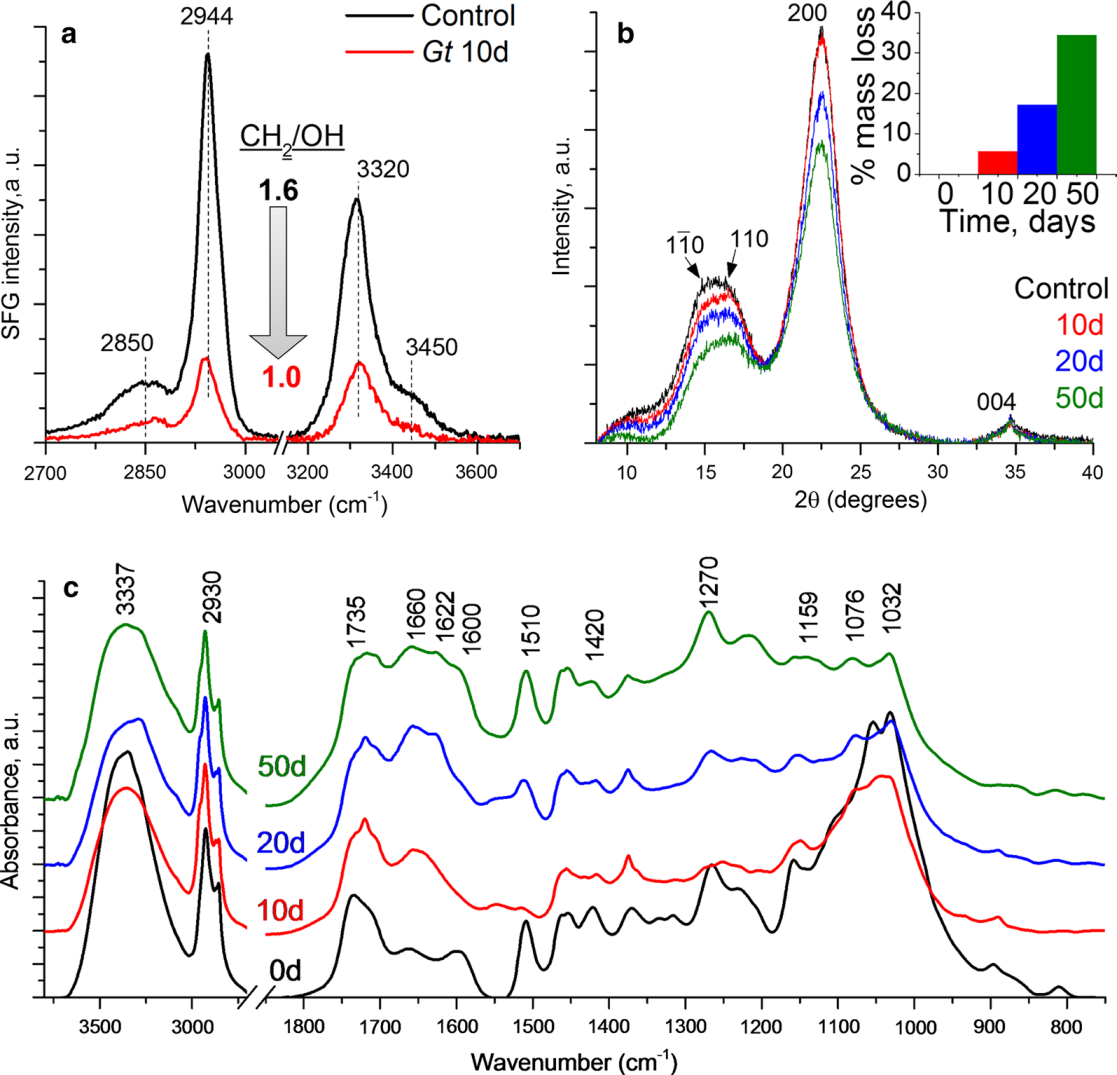
**a** SFG spectra, **b** XRD diffractograms, and **c** ATR-FTIR spectra from the wood wafer surface degraded by *G. trabeum*

**Fig. 4:**
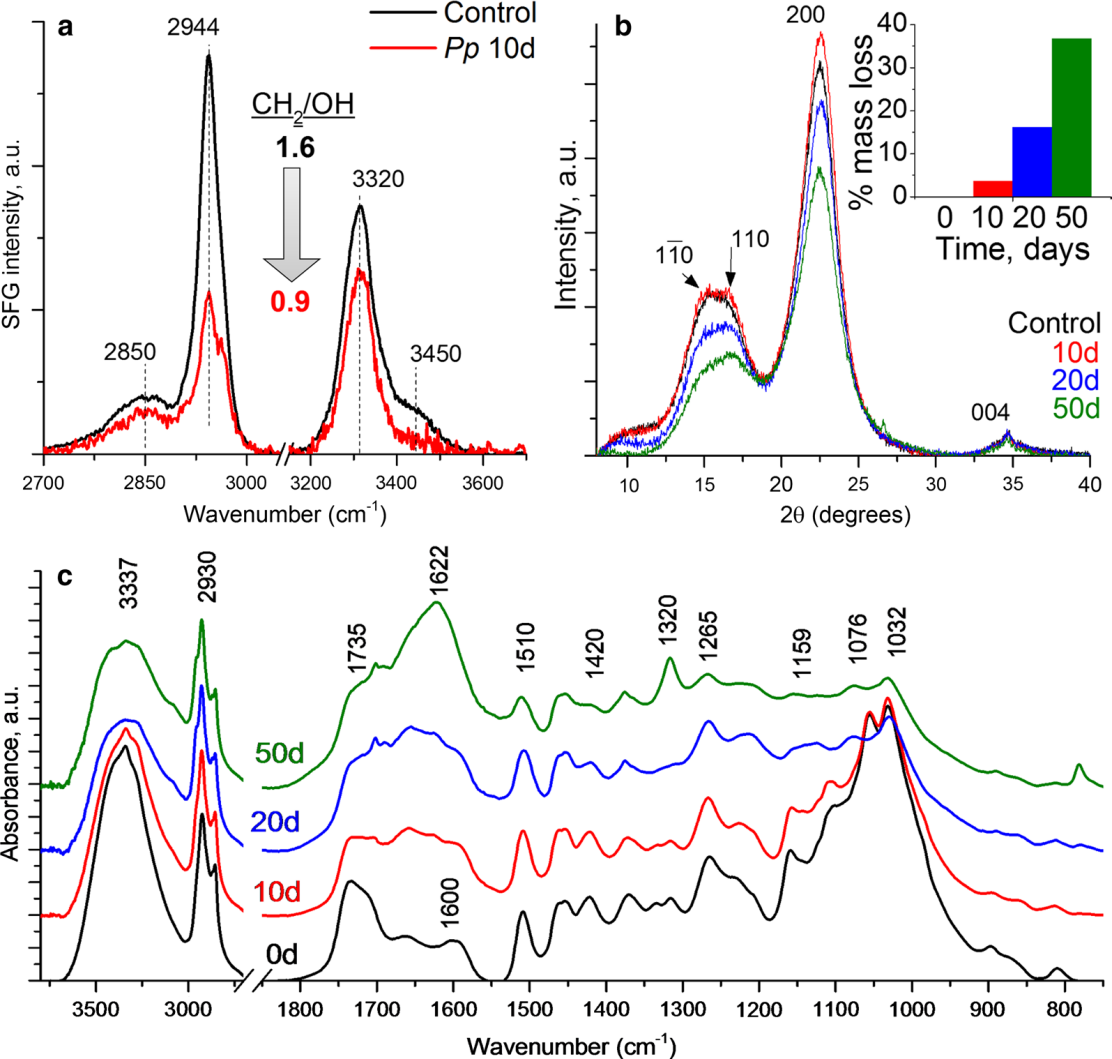
**a** SFG spectra, **b** XRD diffractograms, and **c** ATR-FTIR spectra from the wood wafer surface degraded by *R. placenta*

SFG spectra (Figs. 3a, 4a) collected from control wood wafer surfaces and wood wafer surfaces degraded by either *G. trabeum* or *R. placenta* for 10 days showed a reduction in SFG signal in the decayed samples compared to the control. A typical cellulose SFG signal resembling that obtained from isolated microcrystalline cellulose or cellulose in secondary cell walls from woody tissues [41] was observed in the controls. The alkyl stretch peaks at 2944 cm^−1^ originate from the exocyclic CH_2_ groups of the cellulose chain [41] and the hydroxyl stretch peak at 3320 cm^−1^ is attributed to inter- and intra-chain hydrogen-bonding hydroxyl groups in the cellulose elementary fibrils [42, 43]. The overall SFG signal intensity and alkyl/hydroxyl peak intensity ratio depend on several different structural factors such as the amount of crystalline cellulose, the nature of the crystal structure, and the packing and orientation of the cellulose elementary fibrils [44–49]. After 10 days of incubation with either *G. trabeum* or *R. placenta*, the overall SFG intensity decreased (Figs. 3a, 4a) and the alkyl/hydroxyl intensity ratio also decreased from 1.6 in control to 1.0. The reduction in peak intensity relates to both reduced cellulose elementary fibril packing density, as well as overall reduction in holocellulose [44, 47, 49, 50]. In a previous study by Wang et al. [50], it was also found that a decrease in the SFG peak intensity ratio (CH_2_/OH) correlated with fibrillation of the cellulose elementary fibrils in pretreated biomass samples as observed by TEM. A similar correlation between the reduced CH_2_/OH ratio and lower packing density of cellulose elementary fibrils has been observed for plant cell walls [49, 51].

XRD diffractograms for the *G. trabeum* and *R. placenta* infected wood (Figs. 3b, 4b respectively) suggest a slight decrease in the relative crystallinity (as described by Segal [52]) over the 50 day decay period. It should be noted that the XRD signal reflects the condition of the entire sample depth, while SFG is sensitive only to the surface regions due to the limited IR penetration depth [36]. Because of the type of decay test conducted, surface decay was prominent, while material in the interior of the wafers had more limited or no decay. Thus, the XRD signals were mostly governed in the early decay stages by the “undecayed” interior region of the partially decayed samples, but after 50 days the interior region of the samples also had more advanced decay. The XRD signal with a reduced crystalline to amorphous ratio reflects this in the 50-day sample.

The ATR-IR spectra of the two types of brown rot decayed samples showed drastic changes in the fingerprint region after only 10 days of fungal decay (Figs. 3c, 4c for *G. trabeum* and *R. placenta*, respectively). Changes in the most distinct features between 1500 and 1000 cm^−1^ indicate rapid degradation and depolymerization of the carbohydrate fraction [53]. These results correlate with the observed mass loss in the first period of incubation which typically is associated with the depolymerization of hemicellulose [38]. A decrease in the peak at 1600 cm^−1^ in the first 10 days is associated with vibrational changes in the aromatic ring of lignin, which may indicate either depolymerization, a structural change in lignin, or both. Depolymerization, demethoxylation, and cleavage of the propyl side chain are known to occur during brown rot degradation [13].

### Chelator-mediated Fenton and enzyme treatments

#### SANS analysis of mediated Fenton treatment alone

When used as the sole biomimetic treatment, both Fe-CMF- and Mn-CMF-treated samples (Table 3) displayed a SANS profile similar to that observed when samples were decayed by *G. trabeum* for 18 days (Table 2). However, the scattering feature imparted by the Mn-CMF treatment was less intense when compared to the Fe-CMF treatment, indicating that less oxidation of the wood substrate occurred. The single pulse of CMF treatment as conducted in this research typically results in about 10–15% solubilization of southern pine mass, compared to mass losses of 15–50% when *G. trabeum* decays southern pine in laboratory assays over a 4–8 week period [20, 54, 55]. Subsequent pulses of CMF treatment result in higher mass loss values, and a 4-pulse CMF treatment has been demonstrated to produce an average of 77% mass loss of southern pine [4]. Our goal in this research was not to produce and analyze samples with maximal mass loss, but to compare brown rot attack to CMF treatments as decay progressed in early to moderate stages.

**Table 3:** SANS structural parameters obtained from the unified fit analysis of Fe-CMF- and Mn-CMF-treated samples

Samples	High-*Q*		Mid-*Q*		Low-*Q*
	*R*_*g*_ (Å)	*D* (cylindrical cross-section diameter Å)	*R*_*g*_ (Å)	*D* (Å) (spherical diameter Å)	*P*
0dGt	9.1 ± 1.3	21 ± 3	–	–	3.6 ± 0.1
CMF(Fe)	12.6 ± 0.4	29 ± 1	59 ± 8	152 ± 21	3.3 ± 0.1
CMF(Mn)	11.3 ± 0.4	26 ± 1	70 ± 11	181 ± 28	3.4 ± 0.1
CMF(Fe + Mn)	12.9 ± 0.4	30 ± 1	50 ± 6	129 ± 15	3.2 ± 0.1

When the Fe-CMF and Mn-CMF treatments were combined using the same amounts of 2,3 DHBA and H_2_O_2_, there was no significant difference in *R*_g_ or *P* values across the entire *Q* range (Table 3) compared to Fe-CMF treatment only. This indicates that only limited oxidation of wood occurs when manganese-Fenton reactions were included as opposed to the iron-Fenton reactions alone.

Brown rot decay fungi presumably either pulse low concentrations of CMF reactants into wood over time, or they secrete the reactants at low levels over a sustained period of time. Although multiple pulses, or continual exposure of wood over days to CMF treatment was not conducted in our work to simulate what may occur in advanced brown rot fungal degradation, close similarities in SANS scattering features between brown-rotted wood and CMF treatment were apparent. Redox cycling of specific transition metals occurs via the action of hydroquinone chelators produced by brown rot fungi, and the SANS data support the hypothesis that Fe-CMF treatments (and to a lesser extent Mn-CMF treatment) were able to deconstruct lignocellulose similarly to that observed by *G. trabeum* even in the absence of extracellular enzymes. Prior research suggests that metals such as copper would not be effective in CMF reactions [56].

#### SANS analysis of mediated Fenton treatment or cellulase cocktail

The SANS profile after treatment of pine wood with the Ctec2 enzyme cocktail alone was limited, and similar to that observed after treatment with Mn-CMF (Tables 3, 4). As anticipated, limited change was observed because the SANS data preferentially reflect changes in the spacing between the cellulose crystallites where enzymes are unable to penetrate, and also lignin modification as repolymerization and aggregation occurs. However, the data also suggest that without pretreatment, the enzyme cocktail was unable to efficiently depolymerize or remove hemicellulose from the cell wall or to readily attack the cellulose crystallites. All extracellular enzymes are known to be too large to penetrate the intact cell wall, and thus enzymes are unable to access the cellulose elementary fibril bundles within those walls [16, 24, 11]. Because no lignin-degrading enzymes were present in the CTec2 enzyme cocktail used, the size of the enzymes alone would have limited their access to the interior regions of the wood cell wall and thus limited changes which could have occurred in a limited incubation period.

**Table 4:** SANS structural parameters obtained from the unified fit analysis of enzyme-treated samples

Samples	High-*Q*		Mid-*Q*		Low-*Q*
	*R*_*g*_ (Å)	*D* (cylindrical cross-section diameter Å)	*R*_*g*_ (Å)	*D* (Å) (spherical diameter Å)	*P*
0dGt	9.1 ± 1.3	21 ± 3	–	–	3.6 ± 0.1
Enz	11.1 ± 0.4	26 ± 1	70 ± 13	181 ± 34	3.3 ± 0.1
CMF(Fe) + Enz	12.2 ± 0.4	28 ± 1	46 ± 6	119 ± 15	3.4 ± 0.5
CMF(Mn) + Enz	11.3 ± 0.4	26 ± 1	60 ± 11	155 ± 28	3.4 ± 0.1

When either the Fe-CMF or Mn-CMF treatments were followed by enzyme cocktail treatment, little change in the scattering features was observed compared to the metal-CMF treatments alone. This indicates, relative to neutron scattering effects only, once samples were treated with CMF that additional removal of hemicellulose after treatment by the enzyme cocktail was limited and, as expected, no further modification of lignin by enzyme treatment occurred.

#### SFG, XRD, and FTIR analysis of CMF treatments

**Fig. 5:**
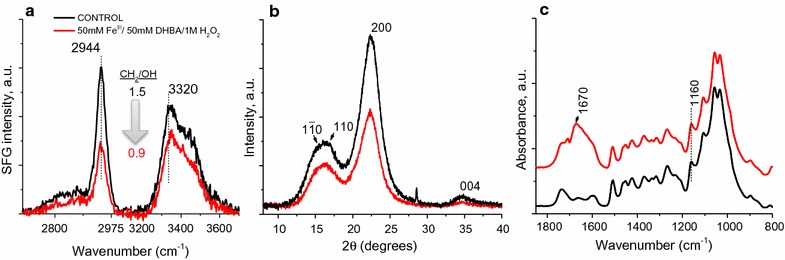
Characterization of pine wood shavings reacted with reactive hydroxyl radical produced during CMF reaction with 50 mM Fe^3+^ by **a** SFG, **b** XRD, and **c** ATR-FTIR

The SFG spectra of iron-mediated CMF treatment (Fig. 5a) show very similar results to that of the wood wafers degraded by brown rot fungi. The overall SFG intensity decreased and the CH_2_/OH intensity ratio also decreased from 1.5 in the control to 0.9 in Fe-CMF (50 mM)-treated wood samples. The lower concentration of CMF treatment with iron also changed the peak intensity ratio (data not shown) but not as dramatically as with 50 mM Fe^3+^. This similarity in SFG spectral changes with CMF and brown rot decay suggests the mechanistic similarity in alteration of cell wall ultrastructure by both the fungus and the mediated Fenton systems (Figs. 3, 4).

For XRD analysis, previous reports have shown that crystalline cellulose is converted to amorphous cellulose during CMF treatment [16, 57]. In our samples, the amorphous cellulose was not removed as crystalline cellulose was converted, except by further degradation of the amorphous cellulose to oligosaccharides. Therefore, an overall reduction in both crystalline and amorphous cellulose would be expected. Figure 5b shows a reduction in signal of both the 200 and 110 peaks and a reduction in intensity of the underlying amorphous signal. When the XRD intensity is normalized with the (200) peak, the valley at 2*θ* = 18° region appears to be slightly higher for the CMF-treated sample than the control sample; however, it should be noted that the small difference in the crystallinity calculated by the Segal method should not be over-interpreted since it can be affected by sample packing and the microfibril packing in the sample [58].

There was limited change noted in the FTIR spectra in the fingerprint region when comparing control vs CMF-treated wood (Fig. 5c). The IR bands in the carbohydrate region (1500–1000) cm^−1^ remain relatively unchanged, which is quite different from the fungal decay spectra (Figs. 3c, 4c). The reason for this is not known, but it could be due to large differences in reaction kinetics and transport of different components in the wood tissues treated by fungus versus CMF. The increase in the band in the 1670 cm^−1^ region which is very close to conjugated carbonyl stretch is unclear, but may reflect the repolymerization of modified lignin, similar to that noted in the brown rot decayed samples.

### Atomic force microscopy (AFM) of brown-rotted wood surfaces

**Fig. 6:**
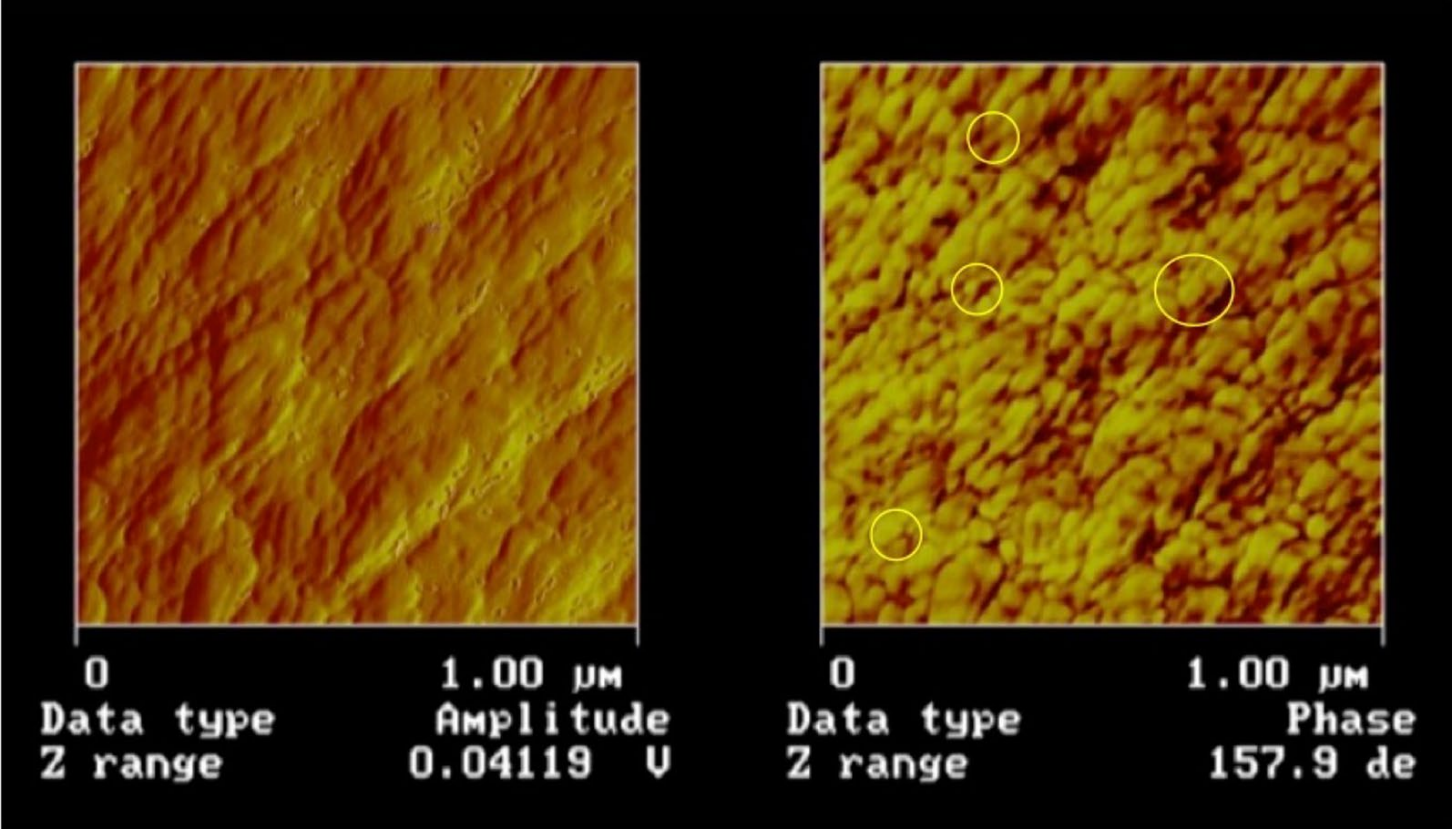
AFM images of *R. placenta* brown rot decayed (8 weeks) S2 cell wall of southern pine wood. *Left* amplitude channel, *Right* phase channel. *Circled regions* show the range in size of the different sized nodules of modified repolymerized lignin in the S2 region of the wood cell wall

The *R. placenta* brown rot decayed samples showed clear evidence of modification of the wood surface at the nanoscale. Unlike the typical smooth or rope-like surfaces seen in wood cell walls of the control samples (not shown) and in the literature [59], the surface of the brown-rotted wood cell walls had a more nodular appearance, with deposits in the range of 25–50 nm in size (Fig. 6). Prior research with heat-treated, and pulped wood has yielded similar types of structural definition as resolved by AFM [60, 61]. Lignin has been reported to be depolymerized, modified, and then rapidly repolymerized during brown rot degradation. We attribute the nodular structures observed to the re-deposition of modified lignin within the S2 wood cell wall layer as hemicelluloses and amorphous cellulose were concurrently depolymerized and removed. Observation of these structural features associated with lignin re-deposition is in agreement with our SANS scattering observations in the mid-*Q* region. It is likely therefore that these features were responsible for changes in SANS scattering observed in that region, as reported in “SANS analysis of fungal hyphae and decayed lignocellulose,” and also observed in the SANS data from the CMF-treated samples.

### Transmission electron microscopy (TEM) analysis

**Fig. 7:**
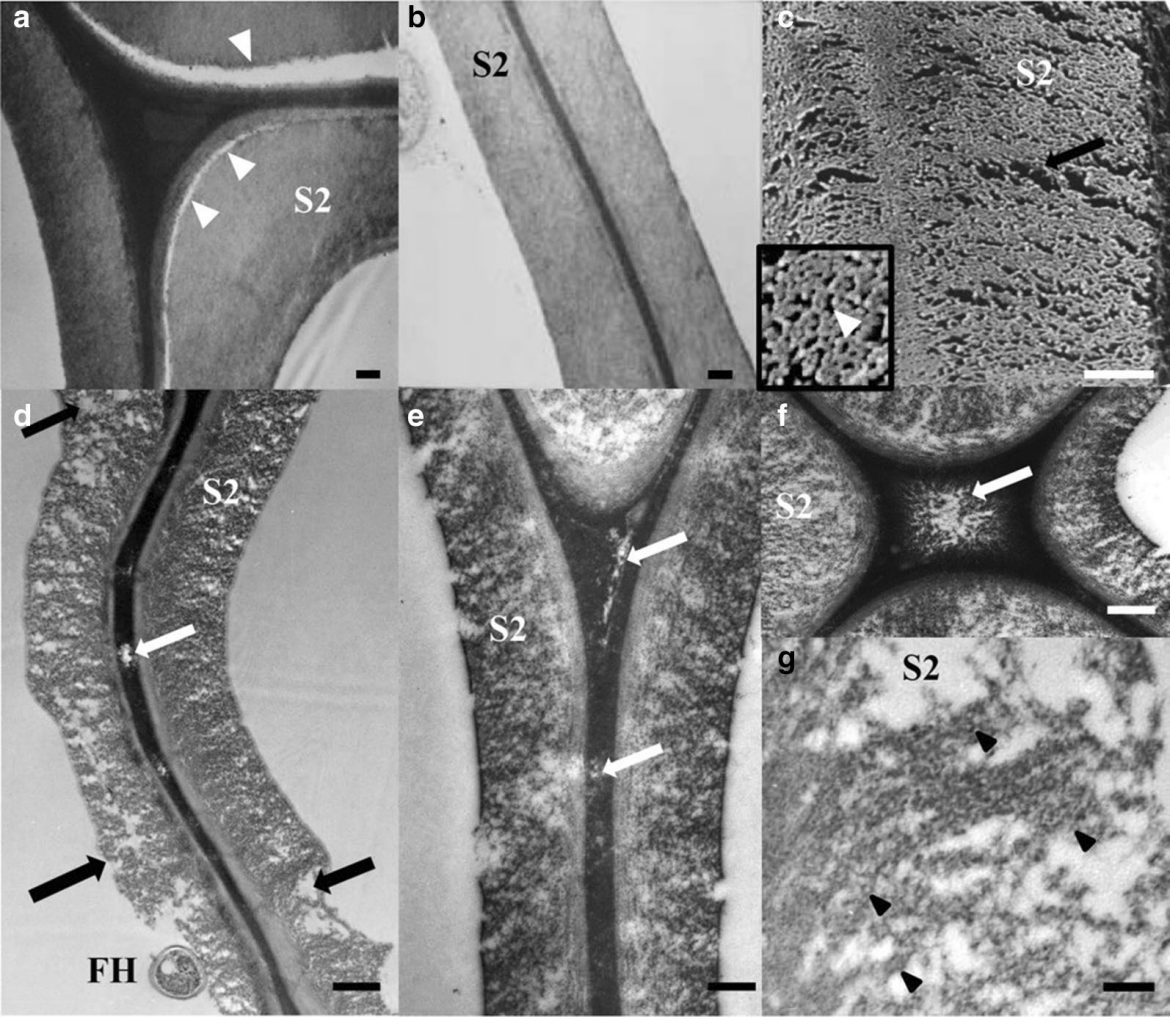
TEM micrographs of pine (**a**, **b**) and TEM and Cryo-SEM of birch (**c**–**g**) wood degraded by *G. trabeum* after 8 and 10 weeks respectively. *G. trabeum* in pine (Fig. 6a, b). At this stage, decay causes weakness between the secondary cell wall layers often resulting in cell wall delamination during sectioning (*arrowheads*). Very little increase in porosity is noted in the brown rot in pine (**c**–**g**). *G. trabeum* attack of the secondary cell walls of birch, in contrast to the pine decay, shows large openings (*black arrows*) developing, even in the lignin rich middle lamellae between fibers (**d**–**f**, *white arrows*). At high magnifications (**g**), individual and well-separated cellulose elementary fibrils (*black arrowheads*) show electron staining of their surfaces. *S2* secondary cell wall, *FH* fungal hyphae. *Bars* **a**–**f** = 0.5 µm, **g** = 10 nm

TEM analysis of pine wood decayed by *G. trabeum* for 8 weeks showed that porosity development in the wood cell wall was limited (Fig. 7a, b). However, some weakening and delamination was observed between the S1 and S2 layers of the secondary cell walls. This contrasts greatly with the increased porosity which occurred, and which is typical of brown rot decay in hardwoods (10 weeks; Fig. 7c–g) including areas within the middle lamellae (Fig. 7d–f). Replicate samples of wood decayed for 10 weeks by *G. trabeum* had in excess of 20 and 30% mass loss in pine and birch, respectively. A major difference between the nanostructure of the cell walls of the fibers in the two wood species is the presence of high levels of guaiacyl lignin in the secondary walls of pine, and syringyl lignin in birch, with the latter having a more open structure. The lack of porosity of the pine after attack by *G. trabeum* suggests that extracellular enzymes would still not be able to access the interior of the cell wall after brown rot attack and the subsequent lack of selection for endocellulase activity may explain the loss of GH6 and 7 endoglucanase genes from brown rot species from the Polyporales and Gloeophyllales [8]. This suggests that a new model for non-enzymatic attack by brown rot fungi should be considered, with the role of enzymatic action at least in softwoods confined to action on oligosaccharides diffusing from the wood cell wall, and/or surface action on the wood lumen surfaces only.

*Gloeophyllum trabeum* attack is typical of brown rot decay in the three fungal orders known to possess the CMF mechanism. As noted earlier, these fungi are known to be capable of depolymerizing cellulose and hemicellulose using a non-enzymatic oxidation process very early in the decay process [2–5]. To permit separation of cellulose elementary fibrils and aggregation of lignin, other components of the wood must be removed or modified. Although both cellulose and hemicellulose are depolymerized in early decay stages, hemicelluloses are preferentially metabolized in the first 2–4 weeks when exposed to *G. trabeum,* and these changes have been reported to account for 20–40% of total mass reduction during this stage [62–64]. Concurrent with depolymerization of lignin, these monomers also undergo biochemical modification, such as propyl side-chain cleavage and demethylation [5, 11–13, 65] prior to repolymerization. In brown rot degradation, our SANS results suggest that the brown rot fungi are able to attack the recalcitrant lignocellulose structure, and modify the cell wall ultrastructure through cellulose fibril compaction as the outer layers of the crystalline elementary fibrils are attacked, and hemicellulose is removed. Concurrently, the redistribution of modified and repolymerized lignin occurs resulting in the formation of lignin aggregates within the cell wall as the decay progresses. These findings are further supported by AFM, SFG and XRD results.

Fungal hyphae alone do not appear to produce SANS features that would interfere with the scattering features observed for wood, or wood undergoing decay. This indicates that SANS can be used as a reliable analysis technique for assessing certain features of lignocellulose degradation by fungi, or by chemical modification in wood that may also be associated with fungal treatments. As noted above, the majority of changes observed in SANS scattering features from wood wafers occurred within 18 days of fungal degradation, with more limited changes occurring in the time period from 18 to 42 days. The changes that occurred suggest a coalescence of cellulose crystallites as depolymerization of the outer crystalline molecular layers occurred, which was confirmed by the changes observed in SFG showing a difference in cellulose packing. SANS proved to be a useful tool to assess these inter-crystallite changes, particularly the changes which resulted in the decrease in the center to center spacing of cellulose crystallites in the cell wall as decayed occurred. Similar changes were observed in both SANS and SFG data when wood was exposed to the CMF reaction, indicating that CMF treatment similarly disrupts the elementary fibril crystallite. XRD data suggest that the CMF system depolymerized cellulose and hemicellulose as well. SANS data in the mid-*Q* region also indicated that particles in the 8–40 nm size appeared. These may correspond to the development of the approximate 25–50 nm size nodules of repolymerized lignin that were observed in AFM after brown rot attack. These observations indicate that aggregation of lignin particles occurs at a larger spatial scale, and one that can be resolved with SANS.

Both the manganese-CMF and the iron-CMF treatments produced SANS profiles similar to that produced by the *G. trabeum* decayed samples. However, the Mn-CMF reaction caused much less modification than the Fe-CMF treatment, and this suggests that manganese plays a less important role in CMF reactions in brown rot fungi than iron. These results indicate that redox cycling of metals occurs via the action of hydroquinone chelators produced by brown rot fungi. The SANS, SFG and other data support that a Fe-CMF, and to a lesser extent Mn-CMF treatment was able to deconstruct lignocellulose similarly to that observed by *G. trabeum* even in the absence of extracellular enzymes.

## Discussion

Taking the results from SANS, SFG, FTIR, and XRD, together with AFM and TEM analysis suggests that lignin as well as both amorphous and crystalline cellulose are depolymerized during decay by brown rot fungi using a CMF mechanism. Porosity of the pine wood cell wall does not increase as decay progresses in early and moderate stages of brown rot, and SANS data indicate that cellulase enzymes will not penetrate and act on the cell wall even as decay progresses, potentially explaining the loss of endoglucanse enzymes from some brown rot lineages. TEM data in pine at resolution of 1 nm (image not shown) confirms the lack of pore development at this level. Because cellulose enzymes are approximately 4 nm along their narrowest point, the structure decayed pine cell wall would not allow enzymes to penetrate. However, in birch (hardwood), more porosity is observed as brown rot attack progresses. This work does not suggest that enzymes would have no role in brown rot degradation of softwood, and it is likely that the limited suite of enzymes produced by the brown rots are secreted to act on oligosaccharides which diffuse from the wood to the lumen, potentially in a staggered mechanism as discussed in recent work [66].

The SANS data also do not preclude that modified lignin re-aggregation may occur in such a way that pores are opened and are then immediately filled with repolymerized lignin. This later process would be consistent with the shrinkage of brown-rotted wood that is observed during brown rot decay processes. Our data suggest that CMF treatment alone is capable of removing considerable amounts of hemicellulose to allow the repolymerized lignin to aggregate, and this finding is supported by earlier literature indicating the hemicellulose sugars are typically the first to be removed in brown rot attack [38, 67]. Despite this removal, cellulase enzyme treatment does not appear to further increase the nanopore access beyond that caused by the CMF treatment.

Overall, our data suggest that CMF treatment mimics the action of the non-enzymatic actions of brown rot fungi as wood decay progresses. The brown rot fungi have been successful for millions of years using a system that our analyses indicate does not open the wood cell wall structure to enzymes. The non-enzymatic removal of cellulosic components while aggregating lignin enables efficient substrate utilization that might be beneficial to species decaying softwood higher in lignin content. Lignin aggregation and maintaining reduced substrate porosity may also limit the access of competing microbes to the resource further enabling brown rot decay species to dominate conifer-rich habitats.

By mimicking the natural action of brown rot fungi, future biorefineries could develop “pretreatment” systems to deconstruct the cell wall without removal of lignin for energy efficiency. This deconstruction could then be followed with enzyme treatment of the soluble polysaccharide fraction using enzymes that have greater activity on the oligosaccharides released. However, CMF pretreatment of softwoods, similar to some chemical and steam treatments which cause similar changes in wood cell wall chemistry, would likely be less effective in allowing enzymes to penetrate the wood cell wall. Repolymerized lignin, which has been demonstrated to be useful as a substitute for phenolic resins [68], would be produced as an additional product. In laboratory studies, we have achieved >75% solubilization of pine wood using CMF reactions, and if the goal is to solubilize the polysaccharide components of softwoods, mimicking the brown rot CMF mechanisms is a straightforward, low-cost, low-toxicity method for solubilization of softwood polysaccharides.
